# Automated multi‐sample DNA extraction for genotyping live *Xenopus* embryos

**DOI:** 10.1002/dvdy.544

**Published:** 2022-10-19

**Authors:** Narmadha Alles, Matthew Guille, Dariusz C. Górecki

**Affiliations:** ^1^ School of Pharmacy and Biomedical Sciences University of Portsmouth Portsmouth UK; ^2^ School of Biological Sciences University of Portsmouth Portsmouth UK

**Keywords:** CRISPR/Cas9, genotyping, *Xenopus*, Zebrafish Embryonic Genotyper

## Abstract

**Background:**

*Xenopus* frogs are used extensively for modeling genetic diseases owing to characteristics such as the abundance of eggs combined with their large size, allowing easy manipulation, and rapid external embryo development enabling the examination of cellular and phenotypic alterations throughout embryogenesis. However, genotyping of mutant animals is currently done either as part of a large group, requiring many embryos, or late in development with welfare effects. Therefore, we adapted the Zebrafish Embryonic Genotyper for rapid genomic DNA extraction from *Xenopus tropicalis* and *Xenopus laevis* at early stages.

**Results:**

Sufficient and good quality DNA was extracted as early as the Nieuwkoop and Faber stage 19 and, importantly, no detrimental effects of the extraction process on the subsequent tadpole development, behavior, or morphology were observed. Amplicons of up to 800 bp were successfully amplified and used for further analyses such as gel electrophoresis, T7 endonuclease I assay and Sanger sequencing.

**Conclusion:**

This method allows rapid genotyping during the early stages of *Xenopus* development, which enables safe identification of mutants. These can be analyzed at early developmental stages or selected for raising without the need for invasive genotyping later, with resource savings and ethical gains in line with the 3Rs principles.

## Key Findings

- Xenopus frogs are used extensively for modelling genetic diseases. However, current procedures for genotyping of mutant animals require many embryos, or are done late in development, which hampers experiments have welfare effects. Therefore, we developed a method allowing rapid genotyping during the early stages of Xenopus development, which has no detrimental effects on the subsequent tadpole growth, with resource savings and ethical gains in line with the 3Rs principles.

## 1 INTRODUCTION

Model organisms are vital contributors to identifying candidate gene variants causing human diseases and in studying their roles. *Xenopus laevis* and more recently the fully diploid frog *Xenopus tropicalis* are widely used in studies of early development and cell biology. *Xenopus* was first recognized as a model organism in the mid 1900s^1^ and quickly discovered to have a great potential for developmental and biochemical studies. Currently, *Xenopu*s frogs are increasingly being utilized to model genetic diseases,^2^ complementing or as an alternative to mice. A key feature of *Xenopus* for its use in genetic studies is the abundance of eggs laid by a female and the ease with which embryos can be genetically manipulated. The power of *X. tropicalis*, when combined with CRISPR/Cas, is the ability to analyze phenotypes even in F0 biallelic mutant animals, which is due to the efficiency of this gene editing technique in the *Xenopus* embryo.^3^, ^4^, ^5^, ^6^ However, the ability to screen mutant or transgenic animals at a large‐scale in a time‐efficient and ethical manner is limited. The strategy commonly applied in such experiments to identify modified pre‐feeding stage embryos, which are mosaic to varying degrees, is to sacrifice and genotype a subset of a group of animals, with the remainder being used for phenotyping. This leads to uncertainty in genotype‐phenotype linkage that needs to be mitigated by using large numbers of embryos. At postfeeding stages, manual tail or toe clipping is used to provide tissue for genotyping.^7^ This creates ethical and resource dilemmas related to raising more tadpoles than are ultimately needed. If individual animals need to be genotyped to strengthen genotype‐phenotype links in F0 studies, it is hugely beneficial that the mutant animals are identified early, before phenotypes first appear. Therefore, it would be of a great advantage to *Xenopus* genetics to develop a method that allows rapid genotyping during the early stages of development, in a manner that is harmless, informs genotype‐phenotype causation and allows tadpoles with the required genotype to be raised to adult frogs.

**FIGURE 1:**
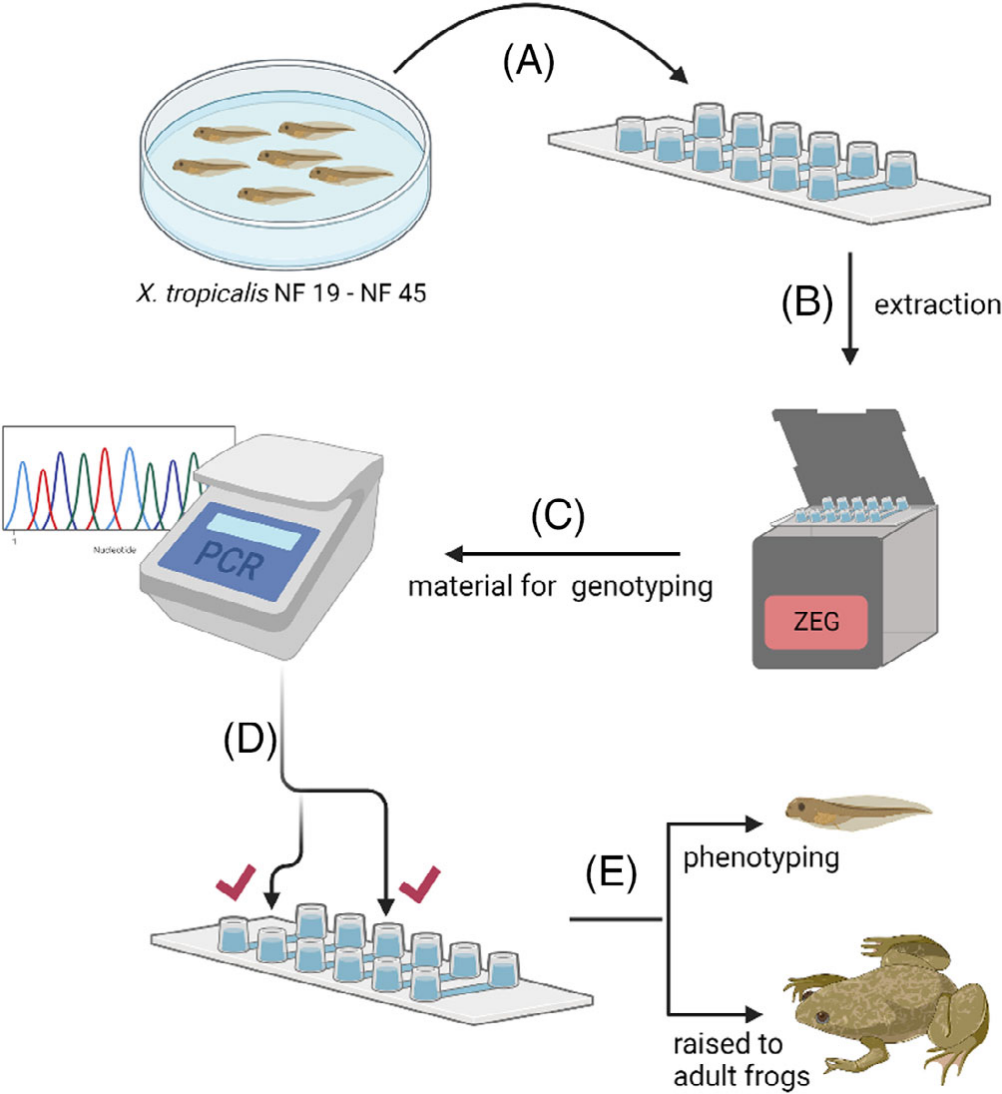
Schematic of the extraction procedure used to effectively genotype embryos. A, Embryos in 0.05X MMR are loaded individually per well of the extraction chip. B, The chip is placed in the ZEG unit and run at 1.8 V for 7.5 min. C, The extracted material is used directly for PCR genotyping and downstream analyses. D, The outcome of the analysis allows identification of embryos of interest, (E) which are then selectively used for phenotyping and/or allowed to develop into adulthood

The Zebrafish Embryonic Genotyper (ZEG) is an automated microfluidic system that extracts genetic material from live zebrafish embryos.^8^ The ZEG is a high‐throughput device that allows extraction of gDNA from 48 to 72 hpf zebrafish embryos in a manner that does not destroy them. The technology uses a microfluidic harmonic oscillation of an animal on an abrasive surface, which generates sufficient genetic material for analysis from 24 individual embryos in 10 min with minimal handling (Figure 1). Such genetic material is suitable for downstream DNA amplification and analyses. Furthermore, its key advantage is that the sampling process is harmless, meaning that the genotypes can be identified and then embryos allowed to develop further into adult animals. For these reasons, we set out to develop a method allowing the use of ZEG for *X. tropicalis* genotyping.

This study describes a detailed methodology in which genomic DNA of *X. tropicalis* is successfully extracted using ZEG from developing embryos as early as NF 19 and evidently does not affect tadpole survival, morphology or behavioral characteristics. We demonstrated that our protocol allows for successful PCR amplification and analysis of regions spanning up to 800 bp. Applicability of this method to *X. laevis* embryo genotyping was also demonstrated.

## 2 RESULTS AND DISCUSSION

**FIGURE 2:**
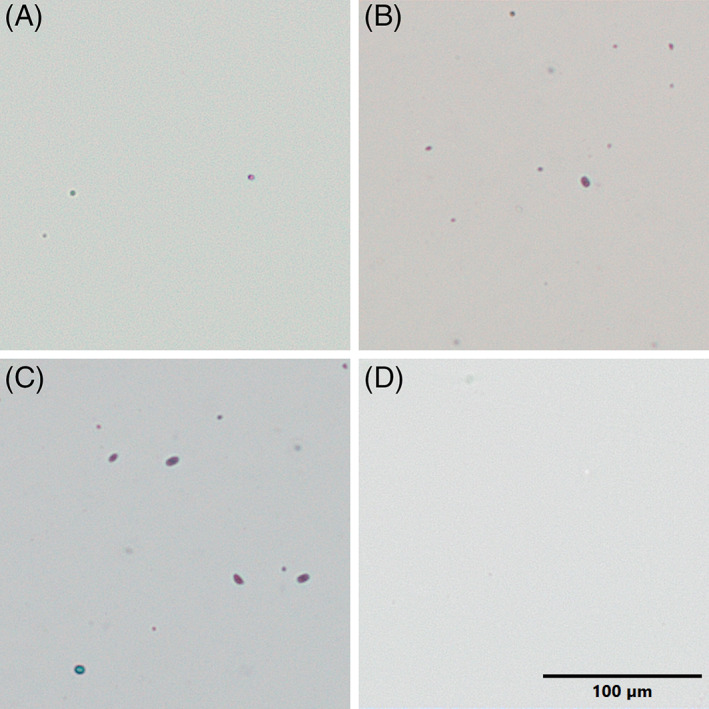
Cellular material extracted using the ZEG. Following ZEG extraction of *Xenopus tropicalis* embryos, selected samples across a range of DNA concentrations were mixed with Trypan Blue (1:1) and imaged; (A) NF 25 ‐ known DNA concentration 32 ng/μL, (B) NF 25 ‐33 ng/μL, (C) NF 19 ‐ 63 ng/μL, and (D) 0.05X MMR (blank). Few Trypan Blue stained (dead) cells and fragments as well as occasional Trypan‐excluding, presumably live cells, are visible in preparations. Scale bar = 100 μm.

To determine the earliest possible developmental stage allowing the safe use of ZEG, samples were extracted from individual wild‐type *X. tropicalis* embryos at NF 19, NF 25, NF 37, NF 42, and NF 45. The DNA concentration in freshly extracted samples was quantified using NanoDrop (Table 1). A high degree of variability in DNA concentration was seen between samples of the same developmental stage, at NF 19: 63 to 100 ng/μL; NF 25: 3 to 116 ng/μL; NF 37: 66 to 91 ng/μL, and NF 42: 7 to 37 ng/μL. In addition, cell counting was attempted using the same samples stained with Trypan Blue (Figure 2). Samples from across the different stages had an average of 27 cells per extracted embryo but again there was a great variability, ranging from 1 to 63. There was no correlation between the DNA concentration and the cell count; for example, some samples with only 1 detectable cell had a DNA concentration between 16 and 45 ng/μL, indicating that cells had been fragmented and therefore the cell count is not an accurate representation of the number of cells extracted using this method nor of the amount of the genetic material present in the extraction medium. Since we found various amounts of DNA to be present in these samples, the integrity and suitability of the genomic DNA isolated using ZEG was investigated in PCR genotyping.

**TABLE 1:** DNA concentration of ZEG‐extracted samples across different stages

Sample	Stage	DNA concentration (ng/μL)		SD	SEM
		Range	Average		
*Xenopus tropicalis*	NF 19M (n = 4)	63‐110	81.5	20.7	10.3
	NF 19 (n = 4)	40‐128	80.2	36.9	18.4
	NF 25 (n = 5)	3‐116	64.0	42.4	16.5
	NF 37 (n = 5)	66‐91	76.8	10.6	4.7
	NF 42 (n = 5)	7‐37	22.9	14.2	6.4
*Xenopus laevis*	NF 22 (n = 6)	9‐36	16.6	10.2	4.2
	NF 24 (n = 6)	6‐66	43.5	23.8	9.7
	NF 27 (n = 6)	9‐45	22.6	8.4	3.4
Zebrafish	72 hpf (n = 15)	7‐81	34.0	26.6	6.9

Abbreviation: NF 19M, NF 19 with vitelline membrane.

**FIGURE 3:**
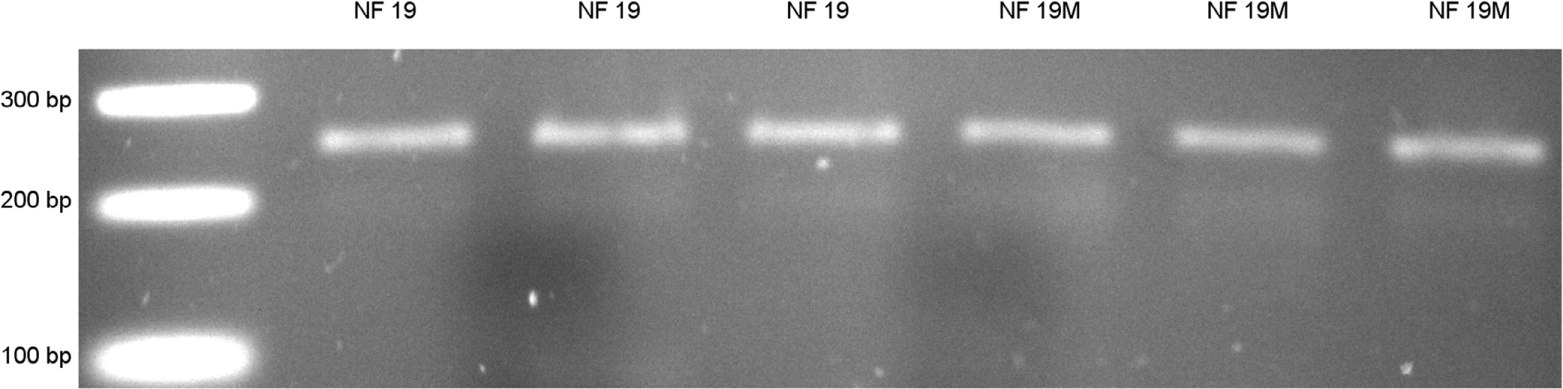
Amplification of ZEG‐extracted DNA from NF 19 *Xenopus tropicalis* embryos. DNA was extracted from NF 19 embryos devitellined (lane 2‐4) and the same stage embryos with vitelline membrane left in situ (M; lane 5‐7). A 260 bp region was amplified successfully as shown resolved on a 1% agarose gel

**FIGURE 4:**
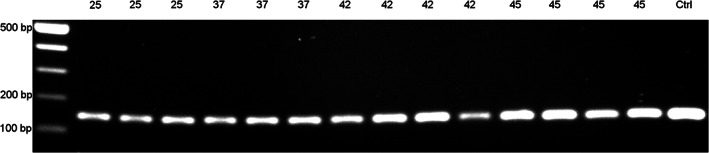
One hundred and fifty base pairs fragments successfully amplified from *Xenopus tropicalis* DNA extracted using ZEG. DNA was extracted from 3‐4 individual embryos at NF 25 (lane 2‐4), NF 37 (lane 5‐7), NF 42 (lane 8‐11), and NF 45 (lane 12‐15). A 150 bp target region was amplified and resolved on a 1% agarose gel. DNA from a sacrificed embryo (NF 25) was extracted by the conventional method and used as a positive control (Ctrl)

To determine whether the presence of the vitelline membrane interferes with the extraction procedure and PCR amplification, NF 19 embryos with an intact vitelline membrane were subjected to extraction alongside devitellined embryos. There was no difference in the genomic DNA amplification between both types of samples (Figure 3), thus showing that amplifiable DNA can be successfully extracted as early as NF 19, irrespective of the presence of the vitelline membrane. Next, PCR amplification was performed across other developmental stages and it resulted in amplicons of expected sizes in all samples. While the band intensity was lower at NF 25, this increased by NF 45, suggesting an improvement in DNA yield (Figure 4). This improvement could stem from the increased genome numbers in the older embryo albeit, due to overall low levels and high variability, DNA concentrations analysis did not support this notion.

**FIGURE 5:**
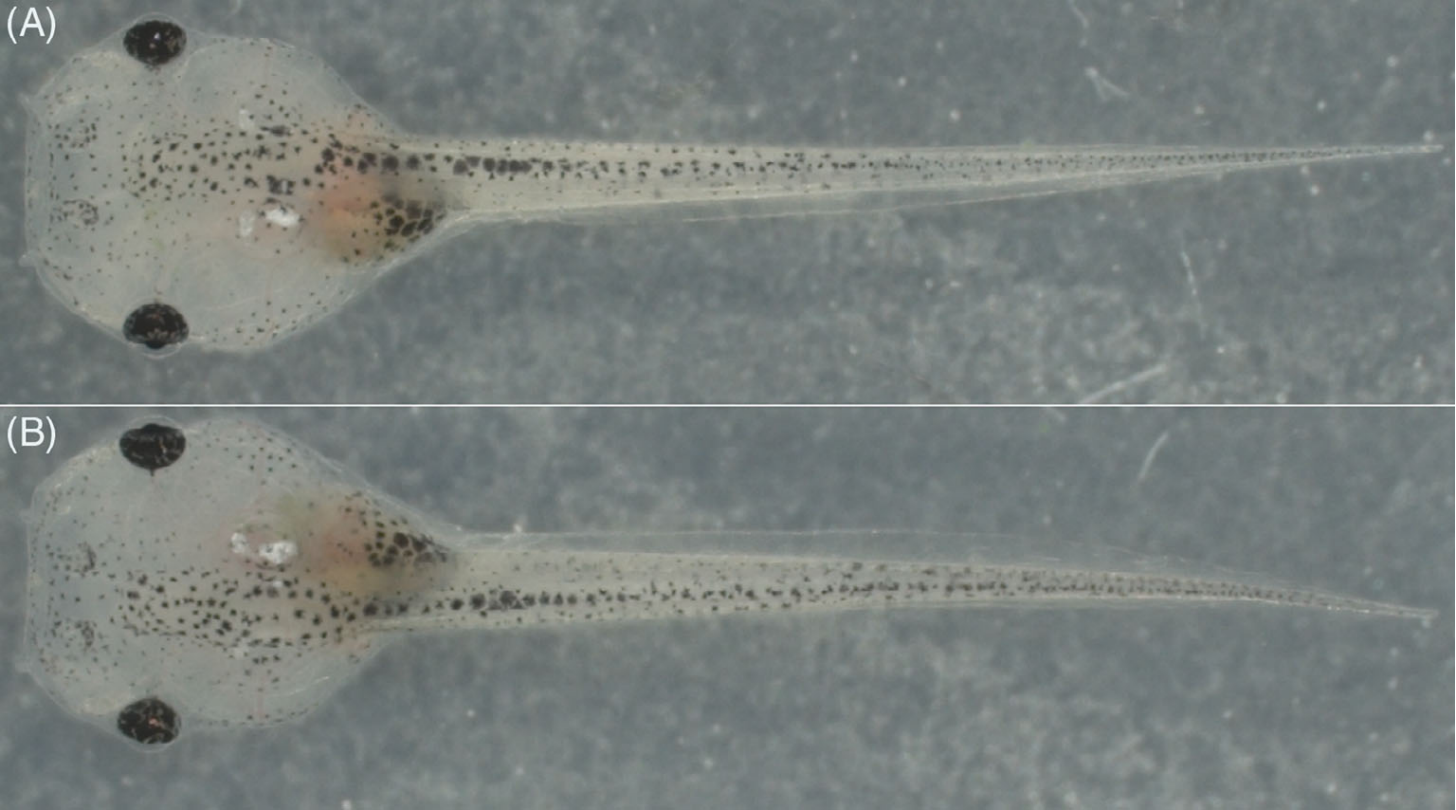
Example images of *Xenopus tropicalis* tadpole. Control, non‐extracted (A) and post‐ZEG extraction (B) at NF 46 are shown. Animals were imaged (25X) and show no visible phenotypic differences

Importantly, all embryos subjected to the ZEG extraction procedure survived, and all that were allowed to grow to free‐swimming and feeding stage (n = 10) showed no obvious behavioral or phenotypic abnormalities (Figure 5). They were indistinguishable from control embryos of the same clutch that were not used in the extraction process.

**FIGURE 6:**
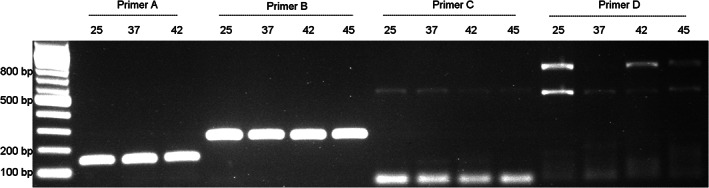
Fragments of different sizes amplified to determine integrity of ZEG‐extracted gDNA. DNA was ZEG extracted at NF 25, NF 37, NF 42, and NF 45, amplified with primer pair A (150 bp), B (260 bp), C (550 bp) & D (800 bp) and resolved on a 1% agarose gel. Contrast was adjusted to reveal the low intensity spurious bands

*X. tropicalis* DNA samples were amplified using primer sets designed to amplify a range of amplicons from 150 to 800 bp. A reduced sensitivity and specificity were observed for the larger amplicons of 550 and 800 bp, as indicated by the faint and non‐specific bands (Figure 6). However, fragments smaller than 260 bp were successfully amplified across all stages analyzed. This suggests that gDNA isolated using this method may be partially degraded into smaller fragments.

**FIGURE 7:**
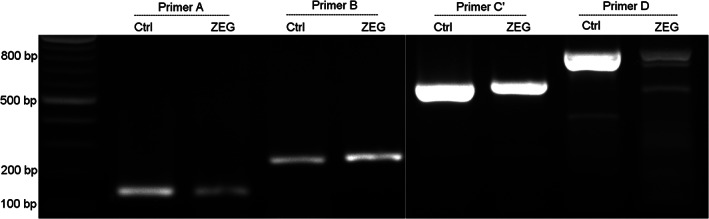
Fragments of different sizes amplified to determine ZEG's sensitivity. DNA was ZEG extracted at NF 37 and amplified with primer pair A (150 bp; lane 3), B (260 bp; lane 5), C′ (580 bp; lane 7), and D (800 bp; lane 9). The samples were resolved on a 1% agarose gel alongside positive control DNA PCR products (Ctrl)

Another possible cause of poorer yield and specificity of the larger amplicons was suboptimal primer design. Indeed, an alternative primer for the 580 bp fragment produced clearly detectable specific amplicons (Figure 7). Amplification of the 800 bp fragment still produced multiple, non‐specific bands.

**FIGURE 8:**
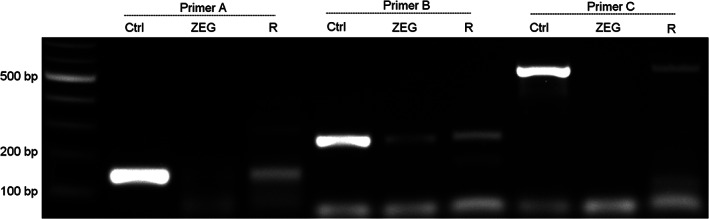
DNA amplification can be facilitated by Restorase. DNA was ZEG extracted at NF 37 and amplified with primer pair A (150 bp; lane 3‐4), B (260 bp; lane 6‐7) and C (550 bp; lane 9‐10). DNA was amplified with either GoTaq G2 Polymerase (ZEG) or Restorase DNA Polymerase (R). The samples were resolved on a 1% agarose gel alongside positive control DNA PCR products amplified with GoTaq G2 Polymerase (Ctrl)

Thus, although the ZEG isolation yields good quality gDNA in most cases and allows rapid genotyping across developmental stages NF 19 to NF 45, the quality of the isolated gDNA can be suboptimal. While a single ZEG isolation was proven to be non‐harmful (Figure 5), it would be better to avoid repeated isolation since this is stressful and potentially could affect embryo viability. Therefore, we investigated whether the ZEG isolation could be combined with recently developed methods allowing reliable amplification of damaged DNA. We evaluated Restorase DNA Polymerase, an enzyme that modifies the damaged site and facilitates amplification of DNA damaged by exposure to acid, alkylating agents, heat, or light.^9^, ^10^ Indeed, Restorase significantly increased PCR amplification of poor quality gDNA templates and in some cases allowed amplification with primers that did not work with standard GoTaq polymerase used in this study (Figure 8).

**FIGURE 9:**
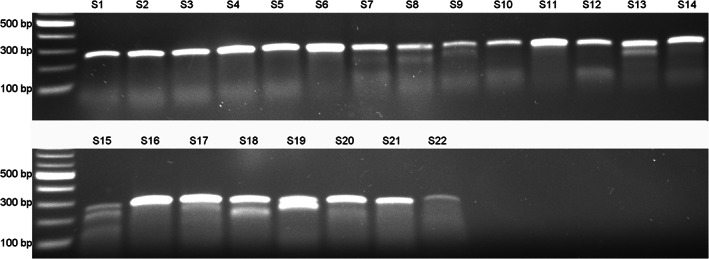
Results of the T7 endonuclease I assay performed to identify mutant animals. *Xenopus tropicalis* embryos (n = 22) were subjected to CRISPR/Cas9 gene editing. DNA was extracted using ZEG at NF 37 and a 260 bp target region was amplified. The amplicons were tested for induced mutations by T7 endonuclease I assay. Samples 8, 9, 13, 15, 18, and 19 showed cut bands denoting potential mismatches as a result of INDELs

**FIGURE 10:**
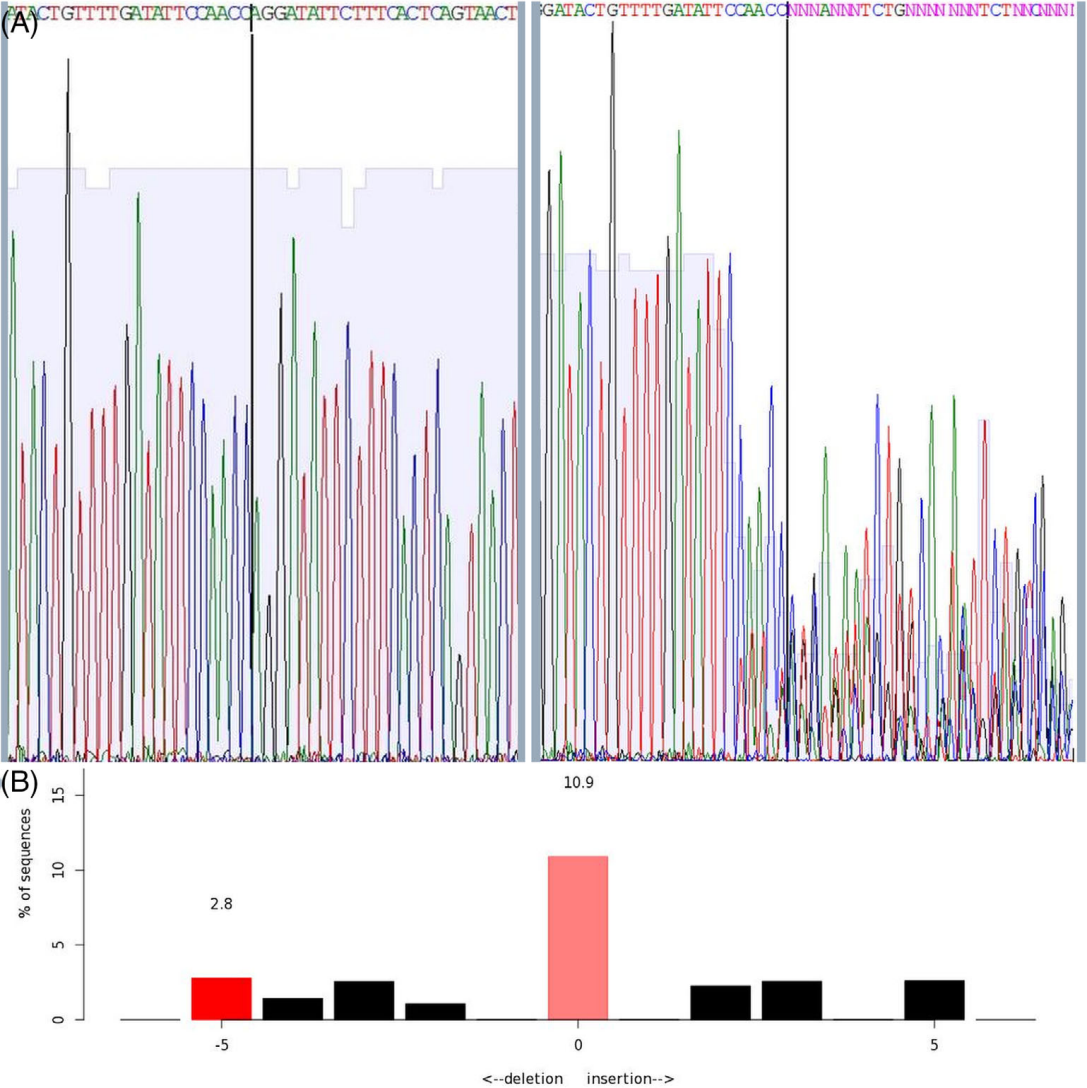
Characterization of INDEL formation using Sanger sequencing and TIDE analysis. Sanger sequencing trace of control (left) and sample 19 (right) ZEG‐extracted DNA. The degradation of the sequence at the expected cut site (black line) results from INDELs caused by the successful CRISPR/Cas‐induced dsDNA break (A). The sequence trace data was input into TIDE to identify the INDELs in the sample (B)

**FIGURE 11:**
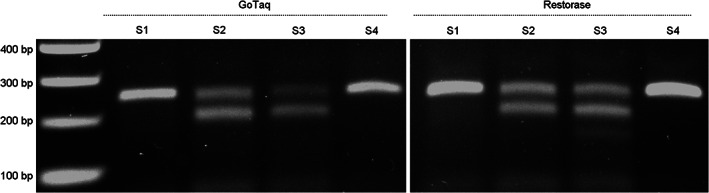
T7 endonuclease I assay performed with Restorase amplified DNA. *Xenopus tropicalis* embryos were subjected to CRISPR/Cas9 gene editing. DNA was extracted using ZEG at NF 37 and a 260 bp target region was amplified with GoTaq G2 Polymerase and Restorase DNA Polymerase. The amplicons were tested for induced mutations by T7 endonuclease I assay. Restorase improved amplification while the mutant samples 2 and 3 were accurately detected with both GoTaq and Restorase amplified DNA

Finally, the ZEG extraction was applied in the course of a gene editing experiment to determine whether mutant animals could be identified. Single guide RNA designed to target a region within the *dmd* gene was co‐injected with Cas9 protein into *X. tropicalis* embryos at 1‐cell or 2‐cell stages and left to grow. gDNA ZEG‐extracted at NF 37 was amplified and tested for successful formation of INDELs using the T7 endonuclease I assay. T7 endonuclease I recognizes two or more mismatches occurring as a result of mutations and cleaves the DNA producing “cut bands” (Figure 9). Following this approach, potential mutant tadpoles were identified, confirmed by Sanger sequencing and characterized by Tracking of Indels by Decomposition (TIDE) analysis (Figure 10). This assay was also performed with ZEG‐gDNA and Restorase and found that Restorase mediated amplification improved the yield (Figure 11). All embryos survived the extraction procedure and are being raised for future analyses and comparisons against those identified as unmodified. Their genotype was later (NF 56‐NF 60) confirmed using the standard tail‐clipping method. Thus, ZEG has a great potential for early, efficient analysis and selection of mutant vs wild‐type embryos, allowing for selective breeding and phenotypic analyses on identified F1s.

**FIGURE 12:**
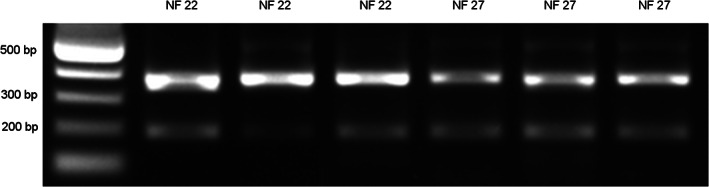
*Xenopus laevis* DNA amplified following ZEG extraction. DNA extracted from devitellined *X. laevis* embryos at NF 22 (Lane 2‐4) and NF 27 (Lane 5‐7), was amplified with primer pair XLA (380 bp amplicon) and the PCR product resolved on a 1% agarose gel. The lower band is non‐specific

In addition, ZEG extraction and PCR amplification were performed on NF 22 and NF 27 *X. laevis* devitellined embryos (Figure 12). This confirmed that *X. laevis* DNA could also be extracted and PCR analyzed using ZEG, further emphasizing the universality of this technique.

Limitations of this new method that were identified include poor priming efficiency and some DNA degradation. Since the yield of intact DNA from young embryos is likely to be relatively low, primers that work at standard DNA concentrations may be insufficiently effective at annealing to produce amplicons when working with *Xenopus* ZEG‐extracted DNA. This problem is not unique to ZEG‐extracted gDNA and can be solved by using further optimized primers. Notably, the combination of the ZEG extraction with amplification using Restorase allowed efficient, non‐destructive analysis of even relatively poor‐quality templates, which otherwise would have to be abandoned. Moreover, owing to the application of ZEG, crispants can be identified as early as the neurula stage. This is both ethically and resource favorable, as embryos containing the desired mutation could be selected to grow and allow for further analysis and downstream experimentation. Our method presents the opportunity to identify F0 biallelic mutant animals very early and to analyze their phenotypes from, at least, neurula stages. An additional gain is that when making lines, the animals to be raised can be identified at early stages and without causing any pain or suffering. Overall, this technique promises resource savings for frog genetics studies alongside ethical gains in line with the refinement principle of the 3Rs.

## 3 EXPERIMENTAL PROCEDURES

### 3.1 Animals

*X. tropicalis* were obtained from the European *Xenopus* Resource Centre, University of Portsmouth and experiments were carried out in accordance with institutional Animal Welfare and Ethical Review Body and the Home Office (UK) Approvals. Adult female *X. tropicalis* were induced to ovulate with 10 IU HCG and boosted with 100 IU HCG the following day. Egg clutches were collected by gentle abdominal massage and fertilized in vitro with cryopreserved sperm.^11^ Frozen sperm was thawed for 30 s in a 37°C water bath, gently mixed with 0.1X Marc's Modified Ringers (MMR): 0.1 M NaCl, 2 mM KCl, 1 mM MgSO_4_, 2 mM CaCl_2_, 5 mM HEPES pH 7.5, 0.1 mM EDTA at a 1:2 ratio and applied onto the eggs. Embryos were cultured at 25°C in 0.05X MMR containing penicillin (5 U)‐streptomycin (5 μg/mL) (Sigma). Staging was carried out according to Nieuwkoop and Faber (NF) staging series.^12^ *X. laevis* embryos, used in some experiments to confirm the universality of this method, were obtained similarly and cultured at 16°C in 0.1X MMR containing penicillin (5 U)‐streptomycin (5 μg/mL).

### 3.2 ZEG extraction procedure

Cellular material from live *Xenopus* devitellined embryos at NF 19 to NF 45 was extracted using the ZEG (InVivo Biosystems) as follows: One embryo was loaded per chamber in a 12 μL volume of 0.05X MMR. The ZEG was run at 1.8 V for 7.5 min. Approximately 10 μL of sample was collected in 0.5 mL PCR tubes and stored at −20°C for subsequent genotyping (Figure 1). Some samples were stained with Trypan Blue added at a 1:1 ratio and the cells were counted using hemocytometer (C‐Chip; Labtech).

Positive control for genetic material (~60 ng/μL) was obtained using the standard procedure involving lysing a single embryo in 60 μL of lysis buffer: 50 mM Tris (pH 8.5), 1 mM EDTA, 0.5% (vol/vol) Tween‐20, 100 μg/mL Proteinase K. The sample was incubated at 60°C for 2 h and 95°C for 10 min for proteinase deactivation, and then centrifuged for 1 min at 750*g* to pellet the debris.

### 3.3 PCR amplification

Primer pairs were designed using Primer3 software^13^ to amplify gDNA regions of different sizes and manufactured by Life Technologies, UK. Primer pair A (150 bp amplicon) Fwd: TGTGTCCCTAGGCAGCCG and Rev: AAAGTACAAATCTGCCCACCTG; B (260 bp amplicon) Fwd: TGAATAGCCGCTGGACAGAA and Rev: CGCTCTGACCTTTGCAAGAT; C (550 bp amplicon) Fwd: CAGGCTTTGTAGTGTGTGGT and Rev: GATCAGCAAGTGTTTCCGCA; C′ (580 bp amplicon) Fwd: CGGACTTTCTGGCTTTTGAC and Rev: TAGGAGGGTCGGTCTCTTCC; D (800 bp amplicon) Fwd: GGGAGTTGTGTGCTGAAGTG and Rev: AATCACACCTACTGCTGCCT; for *X. laevis*, primer pair XLA (380 bp amplicon) Fwd: TAGATAGCAAGCTCTTGGGG and Rev: GCTGCTCTTGCGACTCTTC was used.

PCR reactions (20 μL) were set up as follows: 1X GoTaq G2 Polymerase (Promega), 0.5 μM primers and 5 μL of sample collected using ZEG (2 μL for the positive control). PCR was performed using a Veriti 96‐Well Thermal Cycler (Applied Biosystems) using the following conditions: 95°C for 5 min; 40 cycles of 95°C for 30 s, 30 s at specific primer annealing temperature and 72°C for 1 min; final elongation was at 72°C for 7 min.

### 3.4 Amplification with Restorase DNA Polymerase

To avoid repeated extraction from live embryos, we investigated whether DNA of suboptimal integrity (potentially damaged) extracted using ZEG could still be amplified. For this, amplification was carried out with Restorase DNA Polymerase (Merck). Reactions were prepared according to manufacturer's guidelines; with 1X reaction buffer, 200 μM each of dATP, dCTP, dGTP and dTTP, 1.25 U Restorase, and 5 μL of collected sample. The reactions were preincubated at 37°C for 10 min followed by a further 5 min at 72°C. After the initial denaturation (94°C for 30 s), 0.5 μM of primers was added and PCR amplification was carried out as per the conditions described above.

The resulting PCR products were resolved by agarose gel electrophoresis and analyzed using the G:box F3 (Syngene).

### 3.5 ZEG extraction and analysis of genetically altered embryos

Following in vitro fertilization, embryos were flooded with 0.05X MMR containing penicillin‐streptomycin for 15 min. They were then de‐jellied with 2% (wt/vol) l‐cysteine (pH 7.8) for 5 min and subsequently washed five times in 0.05X MMR. Embryos were microinjected with an injection solution containing 300 ng of single guide RNA targeting the *dmd* gene (synthesized from single‐stranded oligonucleotide taatacgactcactataGGGTGAAAGAATATCCTGGTgttttagagctagaa as described previously^5^, ^14^) and 4 μM Cas9 protein in nuclease‐free water. The injected embryos were supplemented with 0.05X MMR and left to grow at 25°C. ZEG extraction was performed at NF 37 for DNA amplification (with either GoTaq Polymerase or Restorase) and a T7 endonuclease I (NEB) assay was carried out to determine the presence of INDELs. Briefly, 2 μL of amplicon and 2 μL of NEBuffer2 were added in nuclease‐free water to make up a final volume of 19 μL. The reaction was incubated at 95°C for 5 min, cooled to 25°C at a rate of 0.1°C/s and held at 4°C. The sample was incubated with 10 U of T7 endonuclease I at 37°C for 15 mins and the products were resolved on a 1.5% agarose gel. Samples of interest were reamplified, purified (SmartPure PCR Kit, Eurogentec) and INDEL formation was confirmed by Sanger sequencing (Genewiz, UK) and analysis using TIDE software.^15^

## AUTHOR CONTRIBUTIONS

**Narmadha Alles:** Formal analysis (lead); investigation (lead); visualization (lead); writing – original draft (lead); writing – review and editing (equal). **Matthew Guille:** Formal analysis (equal); funding acquisition (supporting); investigation (equal); methodology (supporting); project administration (equal); supervision (equal); writing – original draft (supporting); writing – review and editing (equal). **Dariusz C. Górecki:** Conceptualization (lead); formal analysis (equal); funding acquisition (lead); investigation (equal); methodology (equal); project administration (lead); supervision (equal); writing – original draft (equal); writing – review and editing (lead).
